# Physiotherapeutic Interventions for Upper Cross Syndrome: A Systematic Review and Meta-Analysis

**DOI:** 10.7759/cureus.45471

**Published:** 2023-09-18

**Authors:** Sharmila Chaudhuri, Jasmine Kaur Chawla, Vandana Phadke

**Affiliations:** 1 Amity Institute of Health Allied Sciences, Amity University, Noida, IND; 2 Department of Physiotherapy, School of Allied Health Sciences, Manav Rachna International Institute of Research and Studies, Faridabad, IND; 3 Clinical Research, Indian Spinal Injuries Centre, New Delhi, IND

**Keywords:** rehabilitation, physiotherapy, neck pain, forward head posture, musculoskeletal disorders

## Abstract

Upper cross syndrome is a postural dysfunction that can cause a variety of upper-body musculoskeletal problems. Early detection and physiotherapy can help to prevent further complications. However, no systematic review has evaluated the effect of various physiotherapy intervention strategies to treat this syndrome. Therefore, this study aims to conduct a detailed methodological literature search of the most effective treatment strategies available for the correction of upper cross syndrome.

Prospective human subject studies published in the English language that report the assessment and rehabilitation of upper cross syndrome were included. Clinical trials (randomized and non-randomized) were included when compared to a comparator, control group, and no treatment. The search was limited to human subjects and English-language articles. Outcome measures included craniovertebral angle, kyphotic angle, rounded shoulder, neck or shoulder pain, neck range of motion, electromyographic activity of neck or scapular muscles, and functional limitations. To evaluate the methodological quality of randomized controlled trials, the Cochrane collaboration tool was employed. For non-randomized studies, the Risk of Bias in Non-randomized Studies of Intervention was used. The Grading of Recommendations, Assessment, Development, and Evaluation system was used to rate the effectiveness of the evidence. A random-effect meta-analysis was performed for quantitative analysis to report significant differences based on calculated mean differences, with matching 95% confidence intervals (CIs) whenever possible.

Out of the 34 potentially relevant articles, 18 were included. The postural variables including craniovertebral angle, kyphotic angle, and rounded shoulder showed a significant improvement with the physiotherapy group compared to the no-treatment group (standardized mean difference = -1.78; 95% CI = -2.68 to -0.87; p = 0.0001). Secondary outcomes such as pain and functional limitation showed a significant difference when advanced manual therapy techniques were used compared to conventional therapy (standardized mean difference = -0.71; 95% CI = -1.04 to -0.39; p< 0.0001; and standardized mean difference = -0.57; 95% CI = -1.00 to -0.14; p = 0.009, respectively).

Exercise therapy was found to be beneficial in correcting postural alignment and movement patterns, while manual therapy was found to be similarly effective in pain reduction and functional improvement.

## Introduction and background

Musculoskeletal problems are frequently associated with workplace exposure [1]. Maintaining bad posture over an extended duration can cause postural dysfunction and misalignment [2]. One of the most common consequences of poor posture is upper cross syndrome characterized by tightness of one group of muscles (pectoralis major, upper trapezius, levator scapulae) and weakness of another group of muscles (deep neck flexor, middle, lower trapezius, and serratus anterior) in the upper body [1,2]. The changes can be visualized as the letter X in the upper quadrant of the body [3]. This condition is known as upper cross where the tight set of muscles becomes shortened and overactivated, while the weak group of muscles becomes lengthened and inhibited, resulting in neither group of muscle performing its function efficiently [3].

The prevalence rate varies by employment, with IT professionals showing a prevalence of 67%, students having a prevalence of 37.1%, and laundry employees a prevalence of 28%, among many other professionals, who work in a slouched position for an extended duration [4-7]. In various societies and at various ages, between 11% and 60% of individuals are affected by this syndrome [6,8]. Extended periods of time without treatment can contribute to secondary problems such as impingement syndrome, cervicogenic headaches, shoulder instability due to muscular imbalance, poor joint position sense, and decreased maximal ventilatory ventilation [9]. The symptoms of upper cross syndrome include pain, diminished physical function, and the potential for extended absences from work [10]. This can ultimately put a tremendous economic strain on society [5].

Early detection enables the prescription of the proper countermeasures, preventing the progression of upper cross syndrome. Although numerous studies [9-11] have highlighted various effects of exercise therapy, electrotherapy, muscle energy method, and myofascial release technique, none have provided the optimal course of treatment for upper cross syndrome. The objective of the study is to explore the most effective physiotherapy intervention strategies for upper cross syndrome. This systematic review and meta-analysis adds to the knowledge by identifying the most effective treatments for upper cross syndrome based on the evidence. This will improve performance and physical functioning, maintenance of correct posture, and better quality of life, and will further aid in lowering absenteeism from work.

## Review

Methodology

The study protocol was submitted to the International Prospective Register of Systematic Reviews under the PROSPERO number CRD42021282187. The reporting is according to the Preferred Reporting Items for Systematic Reviews and Meta-Analyses (PRISMA) statement [12].

Data Sources and Searches

An electronic database search was done for randomized and non-randomized clinical trials using PubMed, Scopus, EBSCO Pedro, ScienceDirect, and Google Scholar including the following keywords with combinations using AND/OR operators: upper crossed syndrome, forward head posture, management, rehabilitation, treatment, posture, exercise, manual therapy, neck pain, and shoulder pain. The articles were restricted to those published from inception until February 2023. The search was restricted to research involving human beings and articles published in the English language. Appendix 1 presents the entire search strategy.

Studies about various physiotherapy techniques including exercise therapy, electrotherapy, manual therapy, home exercise programs, and ergonomic advice were included in the analysis. Manual therapy techniques including myofascial release, massage, active release, and muscle energy techniques were also considered. The articles were further screened to ensure that they compared the effectiveness of different physiotherapy/manual techniques to each other or to another control group receiving no treatment, a sham group, or a group receiving other treatment. The studies were included if they reported any one of the outcome measures of forward head or craniovertebral angle, kyphotic angle, shoulder angle, pain, disability, cervical range of motion (ROM), neck muscle strength and endurance, and myoelectric activity of the neck or scapular muscles. These were chosen because all parameters are important to study the recovery of the condition after rehabilitation.

Study Selection

Two reviewers (SC and JKC) separately evaluated the searches, while a third reviewer settled any disagreements. According to the titles and abstracts, all studies that used physiotherapeutic intervention to treat upper cross syndrome were chosen. EndNote reference manager was used to remove duplicate data.

Inclusion and Exclusion Criteria

Population: We included studies involving participants aged 20 to 60 of both genders and from any occupational background who had upper cross syndrome. Only land-based interventions were taken into consideration.

Intervention: All physiotherapy techniques including exercise therapy, electrotherapy, manual therapy, home exercise program, and ergonomic advice were included in the analysis. Manual therapy techniques including myofascial release, massage, active release, and muscle energy techniques were considered for the current analysis.

Comparator: The articles were further screened to ensure that they compared the effectiveness of different physiotherapy/manual techniques to each other or to another control/other treatment/no treatment group.

Outcome measure: The studies were included if they had any one of the outcome measures of forward head or craniovertebral angle, kyphotic angle, shoulder angle, pain due to upper cross syndrome, functional limitation of the neck, cervical ROM, and electromyographic activity of the scapular/neck/shoulder. Studies with additional outcomes such as home exercise programs and ergonomic advice were also included.

The study omitted case series, systematic reviews, theses, dissertations, and studies with duplicate data. It also excluded editorial opinions.

Data Extraction and Quality/Risk of Bias Assessment

Data on the participant’s attributes, such as age, gender, sample size, intervention type, frequency, duration, comparison group, outcome measures, controls, and initial results were taken from each included article. To evaluate the methodological quality of randomized controlled trials and non-randomized studies, the Cochrane collaboration tool and Risk of Bias in Non-randomized Studies of Intervention (ROBINS-I) were used. respectively [13,14]. Based on the possibility of bias, no study was either included or eliminated. SC and JKC independently evaluated the study’s risk of bias, and any potential disagreements were resolved by a third reviewer (VP).

The quality of evidence for outcomes such as craniovertebral angle, kyphotic angle, rounded shoulder, pain, and functional limitation was assessed by the Grading of Recommendations, Assessment, Development, and Evaluation (GRADE) system [13] by the same two independent reviewers (Appendix 2).

They evaluated five characteristics, including study limitation, inconsistency, indirectness, imprecision, and publication bias, which can lower the quality of evidence for randomized trials. Based on this evaluation, the studies were divided into the following four categories: high, moderate, low, and extremely low quality.

Strategy for Data Synthesis

When the trials shared similar patients, interventions, and outcome measures, a meta-analysis was performed using Revman 5.4 (Review Manager 5.4, The Cochrane Collaboration, Oxford, England). Because all data from the included study were continuous, the total effect size was determined using a standardized mean difference (SMD). Each comparison was statistically assessed for heterogeneity if the p-value was <0.05 calculated by chi-square or the I^2^ statistic, used for assessing heterogeneity, was greater than 25%. A random-effect meta-analysis was undertaken [13,15]. If a meta-analysis was not possible, a narrative overview of the findings was presented.

Results

**Figure 1:**
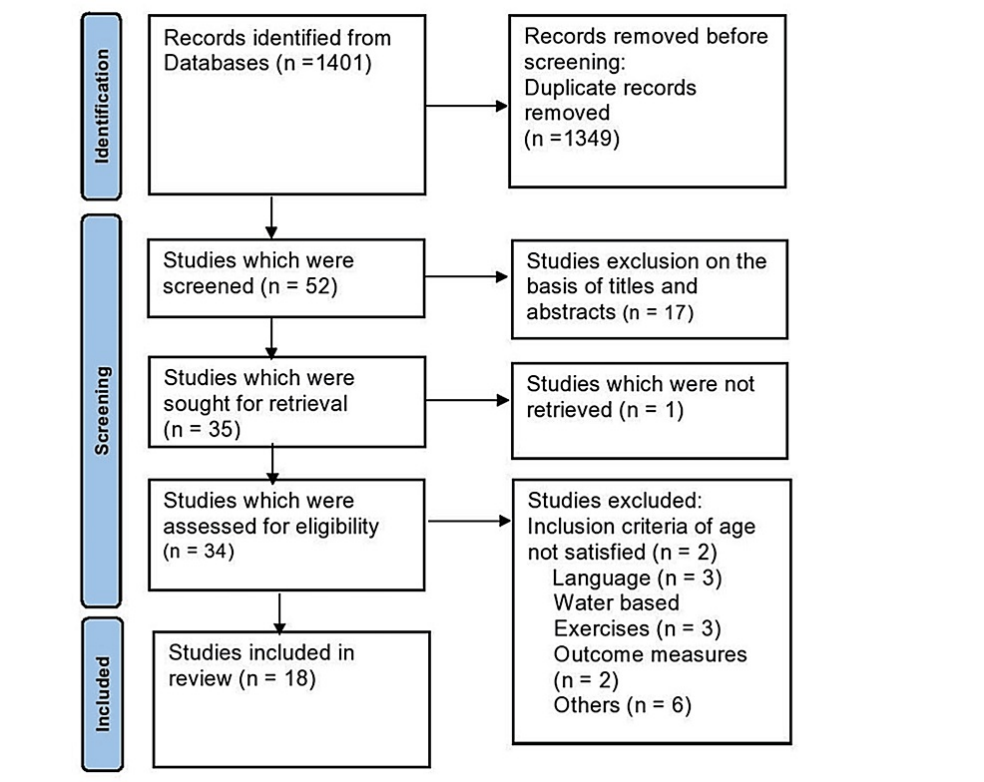
Preferred Reporting Items for Systematic Reviews and Meta-Analyses flowchart.

The literature search yielded 1,401 results. After the exclusion of non-relevant records, 52 studies were found to be eligible for full screening. After screening, 18 studies were included (Figure 1).

Study Characteristics

The review included 18 studies with 846 participants (Table 2) [7-10,16-29]. Ten studies were randomized control trials [7,10,16-23] and eight were non-randomized controlled trials [8,9,24-29]. The lowest mean age reported in the included studies was 20.14 ± 1.71 years and the highest reported mean age was 53.1 ± 6.47 years. Various inclusion and exclusion criteria were reported. Seven studies were conducted in Iran, six in Pakistan, four in India, and one in Thailand. All patients were measured at the baseline before the treatment and after the treatment with the duration of treatment ranging between six sessions for 12 weeks for a duration of 30-70 minutes.

**Table 1:** Characteristics of included studies. N = number of participants; S = sex; M = male; F= female; EG = experimental group; CG = control group; MET = muscle energy technique; RM = repetition maximum; FHP = forward head posture; FSA = forward shoulder angle; TKA = thoracic kyphotic angle; CCEP = comprehensive corrective exercise program; NASM = National Academy of Sports Medicine; TENS: transcutaneous electric nerve stimulation

Trial	Method	Number of participants	Age	Intervention	Outcomes	Results
Seidi et al. (2020) [7]	Randomized controlled trial	N = 24, S = M, EG = 12, CG = 12	EG = 25.3 ± 2.5, CG = 25.4 ± 1.5	EG = CCEP exercises. CG = no treatment. Eight weeks and three sessions per week. Each session = one hour, 10 minutes of warm-up, and five minutes of cool down. The CCEP was designed in three phases, namely, initial, improvement, and maintenance	Postural alignment including FHP, FSA, TKA, scapular movement pattern. Electromyography muscle activity of upper trapezius, middle trapezius, lower trapezius	There was a clinically significant difference between the CCEP and CG in all three outcomes (alignment, muscle activation, and movement pattern)
Karimian et al. (2019) [8]	Semi-experimental	N = 23, EG = 12, CG = 11	EG = 45.2, CG = 44.1	EG = NASM corrective exercise + ergonomic intervention. CG = No treatment. Three sessions per week, with each session lasting for 45 to 60 minutes for 12 weeks	Postural alignment including FHP, FSA, and TKA	Exercises recommended by the NASM and ergonomic changes were a successful rehabilitation program for decreasing kyphosis, forward shoulder, and forward head angles
Piri et al. (2021) [9]	Clinical trial	N = 40, EG = 20, CG = 20	EG = 33.40 ± 2.30, CG = 31.95 ± 1.47	EG = Corrective exercises. 10 minutes of warm up. First phase: self-myofascial release. Second phase: stretching + strengthening. Third phase: dynamic movements were performed, followed by 10 minutes of cool down. Control group = No treatment. Study duration was 12 weeks	Postural alignment including FHP, FSA, and TKA	The findings of this study suggested that female beauticians with upper cross syndrome benefitted from 12-week corrective exercise programs to reduce thoracic kyphosis, forward shoulder, and forward head angles
Yaghoubitajani et al. (2022) [10]	Randomized controlled trial	N = 36, EG1 = 12 EG2, = 12, CG = 12	30–45 years	EG1 = Online: supervised exercises. EG2 = Workplace exercises. Each group received 50 minutes of exercises. Three session per week for eight weeks .CG = No intervention	Pain, sick leave, FHP, FSA, TKA, workability, electromyography	Clinically significant improvements were achieved in the majority of study outcomes after an eight-week corrective exercise program
Arshadi et al. (2019) [16]	Randomized controlled trial	N = 30, S = M, EG = 15, CG = 15	EG = 21.44 ± 2.06, CG = 20.14 ± 1.71	EG = Stretching, strengthening, and stabilization exercises. CG = No treatment. Eight weeks and three of sessions per week, with each session lasting for 50 minutes. Five minutes of warm up and cool down, each at 40% intensity of 10. RM: 70% intensity of 10 RM	Electromyographic activity of muscles upper trapezius, middle trapezius latissimus dorsi serratus anterior, sternocleidomastoid	Significant differences between the pre- and post-treatment characteristics of groups. Corrective exercise helps those with upper cross syndrome regain and maintain balanced muscular function
Ahmad et al. (2019) [17]	Randomized controlled trial	N = 40, M = 18, F = 22. Group A = 20, group B = 20	20-40years	Group A = Myofascial release + self-stretching. Group B = Myofascial release one session per week. The study duration was six weeks	Neck disability index ROM: flexion, extension, left side flexion, right side flexion, right rotation, left rotation, pain	A more effective treatment for upper cross syndrome was self-stretching combined with myofascial trigger point release than myofascial trigger point release alone
Rana et al. (2020) [18]	Randomized controlled trial	N = 60	Group A = 25.17 ± 4.18, Group B = 25.37 ± 5.04	Group A = Stretching + strengthening exercises + hot packs. Group B = Stretching + strengthening exercises + muscle energy technique. Three sessions per week for eight weeks	Neck disability index, pain duration of 4–12 weeks	Upper cross syndrome can be treated with both conventional therapy and the muscle energy technique, although it was determined that the muscle energy technique was preferable to conventional therapy
Rayjade et al. (2020) [19]	Randomized controlled trial	N = 60, M = 28, F = 32. Group A = 30, Group B = 30	21–40 years	Group A = Kinesio Tape + strengthening +stretching exercises + hot pack. Group B = Interferential therapy + hot pack + strengthening exercises. Three sets of 10 repetitions for a total duration of two weeks. Deep neck flexors, middle and lower trapezius, and serratus anterior strengthening. Stretching of pectoralis major and minor muscles. Five repetitions for 30-second holds	Postural alignment including FHP, FSA, and pain	There was a significant reduction of pain and neck disability in the patient population. In comparison to conventional therapy, patients with upper cross syndrome may benefit from Kinesio taping to ease their pain and improve their neck angles
Gilani et al. (2020) [20]	Randomized controlled trial	N = 40. Group A = 20, Group B = 20	Group A = 42.75 ± 11.13, Group B = 40.50 ± 9.14	Group A underwent eccentric muscular energy technique with cervical segmental mobilization. With cervical segmental mobilization, a slow stretch was administered for 6–60 seconds. Group B received static stretches for the upper trapezius, levator scapulae, and pectoralis major muscles. TENS was administered to both groups for 10–20 minutes at a high frequency and low intensity of 50–100 Hz at a pulse width of 50–200 seconds along with 10 minutes of infrared light	FHP, pain duration <3 month to more than a year, neck disability index, and cervical ROM	Upper cross syndrome symptoms were effectively managed by combining both treatment modalities with TENS, IR/hot pack therapy, and mobilization. No combination outperformed the others
Amjad et al. (2020) [21]	Randomized controlled trial	N = 120. Group A = 60, Group B = 60	Group A = 44.4 ± 9.42, Group B = 45.75 ± 9.33	Group A = McKenzie traction + general exercises. Group B = General exercises + TENS for eight weeks, three times/week	Oswestry disability index, neck ROM. Flexion, extension, side flexion, and rotation	Exercise and McKenzie treatment both reduced neck pain, but McKenzie treatment was superior to routine exercise
Nitayarak and Charntaraviroj (2021) [22]	Randomized controlled trial	N = 40, EG = 20, CG = 20	EG = 20.26 ± 1.20, CG = 20.15 ± 1.27	EG = Scapular stabilization was performed three times, with each repetition lasting 10 seconds. CG = No treatment. Three days per week for four weeks under supervision	Postural alignment including FHP, FSA, and TKA. Scapular muscle strength, pectoralis minor length	The forward head and shoulder posture, pectoralis minor muscle flexibility, and scapular muscle strength were improved by a four-week scapular stabilization exercise program
Mahmood et al. (2021) [23]	Randomized clinical trial	N = 60. Group A = 30, Group B = 30	Group A = 31.50 ± 6.388, Group B = 32.60 ± 5.55	Group A received routine physical therapy treatment, while experimental group B had instrument-assisted soft tissue mobilization. For four weeks, three timesp er week	Neck ROM (extension, flexion, neck right bending, and neck left bending) pain	Routine physical therapy combined with instrument-assisted soft tissue manipulation was highly effective in treating upper cross syndrome symptoms such as pain and restricted range of motion in the affected neck and surrounding muscles
Ali et al. (2017) [24]	Randomized comparative study	N = 52	20–50 years	Group A = MET, Group B = Stretching. Three sessions per week for 16 sessions	Neck disability index ROM: flexion, extension, side bending, and rotation	Compared to stretching exercises, MET demonstrated greater progress in reducing pain, enhancing range of motion, and enhancing function
Javesi et al. (2019) [25]	Quasi-experimental	N = 24, S = F, EG = 12, CG = 12	18–25 years	EG = Exercise with physio ball. CG = No treatment. Six weeks	Postural alignment including FHP, FSA, TKA, and chest expansion	The decrease in thoracic kyphosis, forward head, round shoulders, and chest expansion indicate the efficacy of the exercise program
Abdolahazad and Danesh mandi (2019) [26]	Experimental study	N = 30, S = F, EG = 15, CG = 15	18–25 years	EG = Exercises were given in four phases inhibition, stretch, activation, coherence. CG = No treatment. Eight weeks and three sessions per week. Sessions lasted from 30 to 70 minutes	Postural alignment including FHP, FSA, and TKA	The study showed the benefits of NASM-based exercise for muscle balance, reversing head forward posture, rounded shoulders, and thoracic kyphosis
Risalda et al. (2021) [27]	Comparative study	N = 35, Group A = 18, Group B = 17	18–35 years	Group A = active release technique and static stretching along with hydrocollator pack. Active release technique 8–10 minutes, static stretch 15–30 seconds, 2–3 repetitions. Group B = traditional physiotherapy, including hydrocollator packs, levator scapulae and pectoralis major stretches, as well as lower trapezius, deep neck flexor, and rhomboid muscle strengthening. Six sessions of therapy were administered with 10–15 repetitions	Neck disability index, cervical ROM, and pain	In terms of the treatment protocol for the pain reduction, active release technique among upper cross syndrome patients demonstrated favorable trends and was advantageous for patients when compared to conventional physiotherapy
Zad and Patil (2021) [28]	Experimental study	N = 52	22–28 years	10 minutes of warm up + stretching + strengthening exercises + 10 minutes of cool down + ergonomic advice. Five sessions per week for three weeks	Postural Alignment including FHP, Neck disability index, Pain.	Janda’s technique resulted in a considerable decrease in the indexes for pain, craniovertebral angle, and neck impairment
Sasun et al. (2022) [29]	Experimental study	N = 80. Group A = 40, Group B = 40	Group A = 22.95 ± 1.66, Group B = 22.85 ± 1.81	Group A = Myofascial rollers + conventional therapy; 20 minutes four session per week. Group B = Post-isometric relaxation technique + conventional therapy for 20 minutes; four session per week	Pain, plumb line	The study proved that myofascial rollers worked better than post-isometric relaxation treatments to reduce pain intensity and rectify postural abnormalities in dentistry students with upper cross syndrome

**Figure 2:**
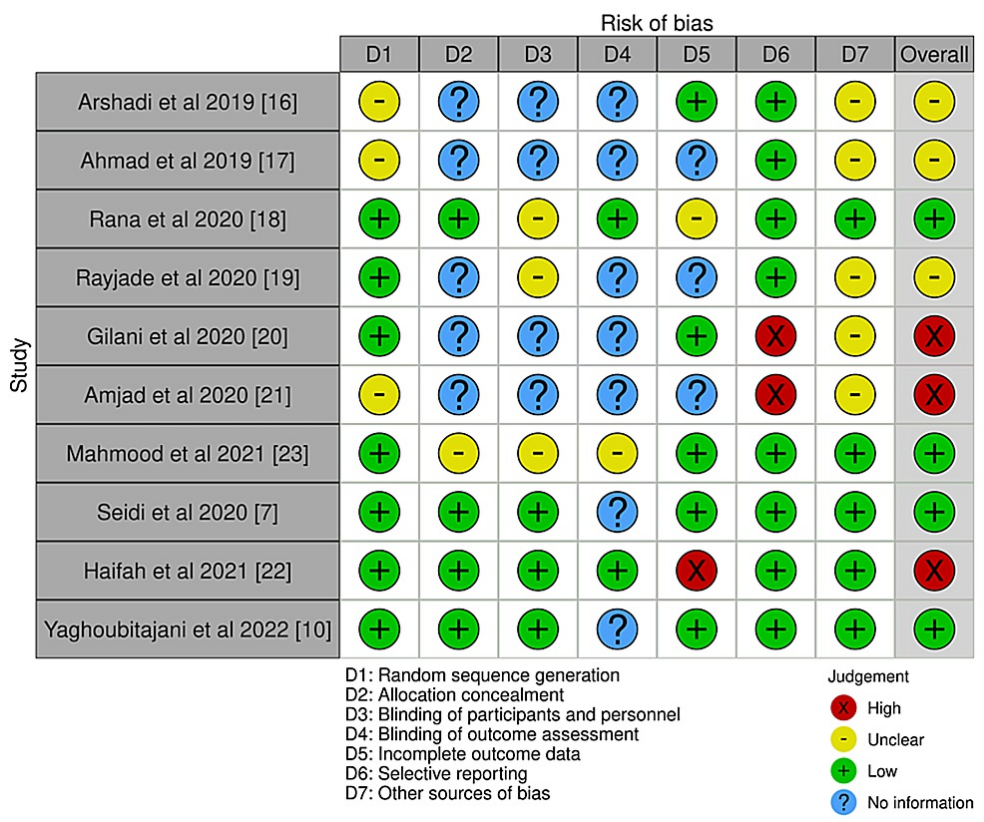
Risk of bias assessment using the Cochrane Risk of Bias tool for randomized controlled trials. Risk of Bias VISualization [30].

**Figure 3:**
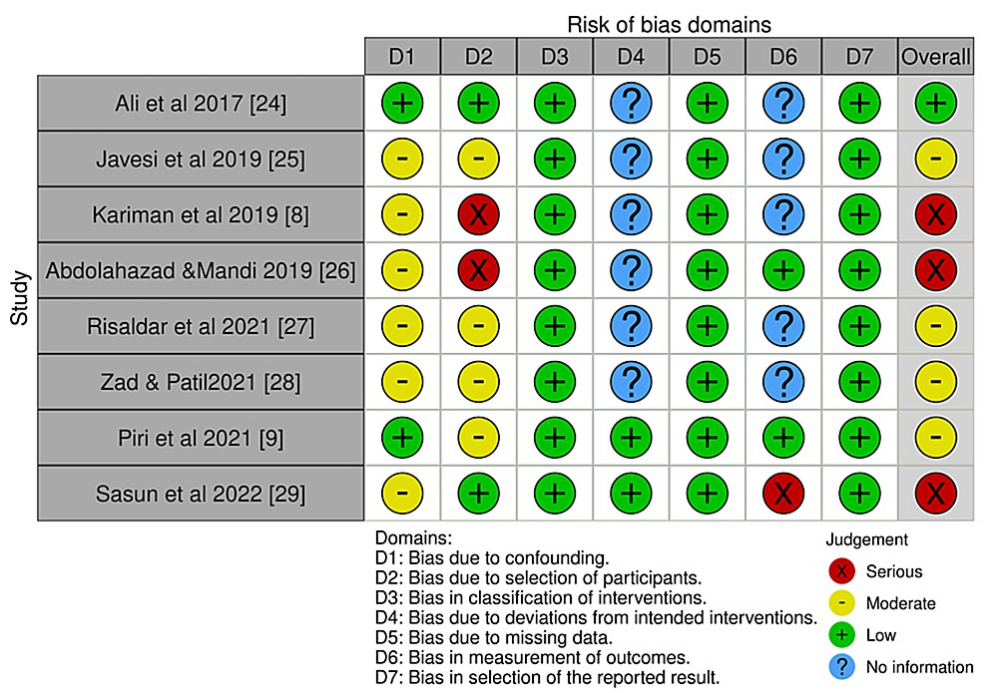
Risk of bias assessment using Risk of Bias in Non-randomized studies of Intervention for non-randomized studies. Risk of Bias VISualization [30].

The Risk of Bias VISualization (ROBVIS) [30] was used to show the results of the quality assessment of intervention studies (Figures 2, 3). The risk of bias assessment was frequently high to moderate and ambiguous in many of the investigations. In the randomized controlled trials, the process of randomization was not explained in many of the studies [16,17,19-21]. The detection bias could not be ruled out as it was not possible to blind the outcome assessor. The risk of bias increased due to the existence of confounders [8,25-29] and the subject selection procedure [8,9,25-28] in non-randomized control trials, with a single study having a low risk of bias [24].

Primary Outcome

Postural parameters: A meta-analysis of randomized controlled trials and non-randomized controlled trials was done separately for postural parameters, and the studies were divided into three subgroups which included craniovertebral angle, rounded shoulder, and kyphotic angle.

**Figure 4:**
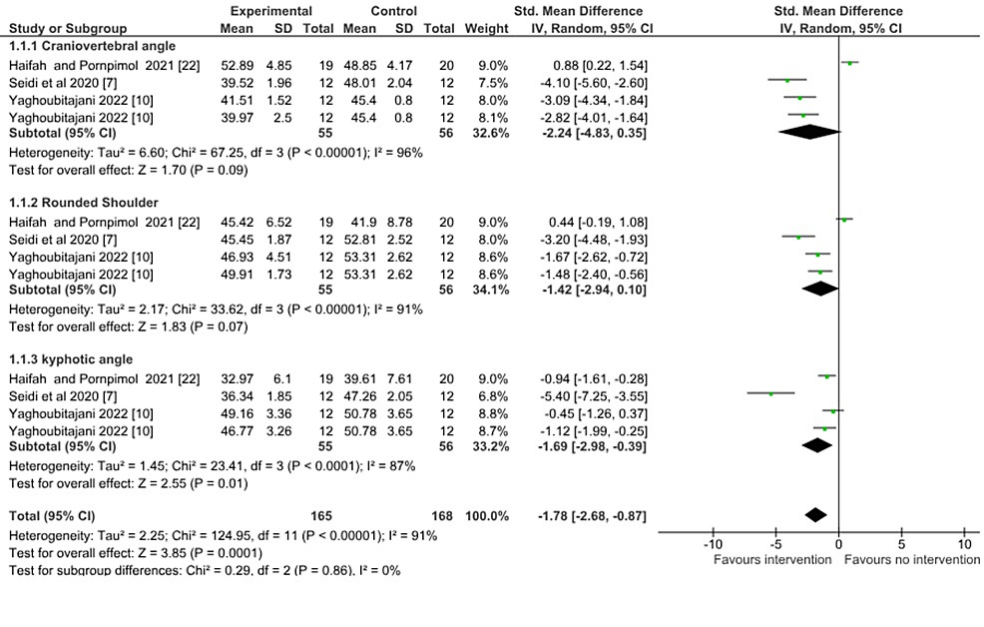
Forest plot of physiotherapy (exercise therapy) versus control Group (no therapy) for the outcome of postural parameters (randomized controlled studies). SD = standard deviation; CI = confidence interval; Chi^2^ = chi-square statistic; P = p-value; df = degree of freedom; I^2^ = heterogeneity I^2^ statistic; Z = z statistic

For randomized controlled trials, three studies [7,10,22] evaluated physiotherapy (exercise therapy) versus no therapy on postural parameters (craniovertebral angle, kyphotic angle, shoulder angle) post-intervention. Meta-analysis showed a significant effect favoring physiotherapy on postural parameters (SMD = -1.78; 95% confidence interval (CI) = -2.68 to -0.87; p = 0.0001). There was evidence of high heterogeneity with I^2^ of 91% in this comparison (Figure 4).

**Figure 5:**
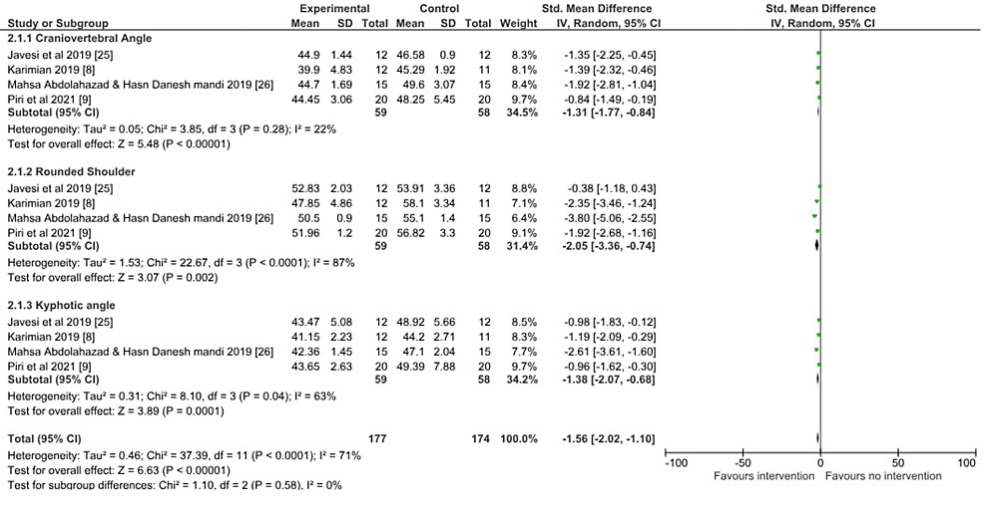
Forest plot of physiotherapy (exercise therapy) versus control group (no therapy) for the outcome of postural parameters (non-randomized controlled studies). SD = standard deviation; CI = confidence interval; Chi^2^ = chi-square statistic; P = p-value; df = degree of freedom; I^2^ = heterogeneity I^2^ statistic; Z = z statistic

For non-randomized controlled trials, four studies [8,9,25,26] evaluated physiotherapy (exercise therapy) versus no therapy on postural parameters post-intervention Meta-analysis showed a significant effect favoring physiotherapy on postural parameters (SMD = -1.56; 95% CI = -2.02 to -1.1; p < 0.00001). There was evidence of considerable heterogeneity with I^2^ of 71% in this comparison (Figure 5).

Secondary Outcomes

**Figure 6:**
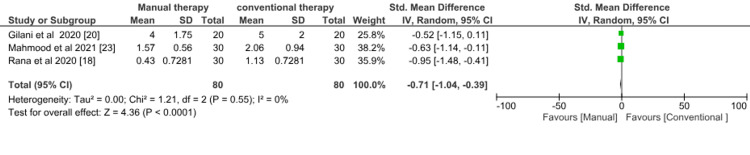
Forest plot of physiotherapy (manual therapy) versus physiotherapy (conventional therapy) for the outcome of pain. SD = standard deviation; CI = confidence interval; Chi^2^ = chi-square statistic; P = p-value; df = degree of freedom; I^2^ = heterogeneity I^2^ statistic; Z = z statistic

Pain: Three studies [18,20,23] evaluated manual therapy versus conventional therapy on pain. Meta-analysis showed a significant effect favoring manual therapy (SMD = -0.71; 95% CI = -1.04 to -0.39; p < 0.0001). There was evidence of low heterogeneity with I^2^ of 0% (Figure 6).

**Figure 7:**
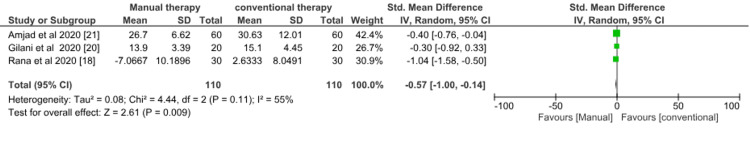
Forest plot of physiotherapy (manual therapy) versus physiotherapy (conventional therapy) for the outcome of functional disability. SD = standard deviation; CI = confidence interval; Chi^2^ = chi-square statistic; P = p-value; df = degree of freedom; I^2^ = heterogeneity I^2^ statistic; Z = z statistic

Functional disability: Three studies [18,20,21] evaluated manual therapy versus conventional therapy on disability. Meta-analysis showed a significant effect favoring manual therapy (SMD = -0.57; 95% CI = -1.00 to -0.14; p = 0.009). There was evidence of considerable heterogeneity with I^2^ of 55% in this comparison (Figure 7). Heterogeneity in the above studies can be explained based on the population and variation in the rate, frequency, type, and duration of the treatment.

Discussion

The objective of this review was to analyze and understand the best possible physiotherapy treatment strategies for upper cross syndrome. The current poor to very low-quality evidence suggests that exercise therapy can affect postural imbalance in upper cross syndrome. Further, the inclusion of manual therapy in treatment protocols may help in improving the physical outcomes of neck pain, mobility, and functional limitation. As good-quality studies are not available, future studies of high quality are recommended to increase the body of evidence and deliver more reliable findings.

This systematic review was unique in many ways in terms of defining scope, inclusion criteria, use of alternate search methods, and constraints. An earlier systematic evaluation focused on the impact of different exercise programs on upper cross syndrome and excluded other physical therapy treatment methods such as manual therapy [11]. This is the first systematic review to discover physiotherapy intervention options for treating upper cross syndrome. Several physiotherapeutic strategies for treating and preventing upper cross syndrome have been shown to be effective, but none have explicitly declared which treatment strategy is more effective [31].

The study corroborates the previously reported literature that highlighted the effectiveness of exercise therapy protocols for the correction of postural imbalance associated with upper cross syndrome. Additionally, scapular stabilization exercises when performed for the middle, lower trapezius, serratus anterior, and rhomboid showed a significant improvement in postural alignment, pectoralis minor length, and scapular muscular imbalance [22].

Exercises recommended by the National Academy of Sports Medicine combined with ergonomic intervention was another successful therapy approach. This has four stages, namely, lengthening, activating, inhibiting, and integrating, and it was successful in treating upper cross syndrome [8,26].

Furthermore, a comprehensive corrective exercise program was used for neuromuscular rehabilitation to change motor patterns. Activating the weak muscles and relaxing the tight muscles were accomplished via electromyography biofeedback. The objective was to promote muscular motor activity by incorporating motor learning principles into early therapy. The movement that is produced by the human body is the result of intricate interactions between the articular, muscular, and neurological subsystems. Upper cross syndrome can be managed well using a multimodal strategy that simultaneously emphasizes posture, movement pattern, and muscle activation [7].

Online-supervised corrective exercise in the workplace was found to produce better results than exercising alone or without any guidance [10]. When used together in combination, Kinesio taping and exercises were found to be more effective at reducing pain than conventional therapy. By inhibiting the pain gate pathway, proprioceptive input from the Kinesio taping helped to block the patient’s nociceptive signal. To reduce pain and enhance function, electrotherapy techniques such as transcutaneous electrical stimulation, interferential therapy, and hot packs can be used with exercise treatment [19]. Moreover, this review identified only one study that used physioball as a tool for corrective exercises as it engaged more muscles overall, including the muscles in the shoulder girdle, lower limbs, and trunk [25].

It was observed that many manual therapy approaches, including myofascial release, active release technique, and muscular energy method, were useful in the treatment of this syndrome. The short-term benefits of manual therapy over standard therapy have been shown in several studies, and the effects on pain and functional outcome markers have been discussed [17,20,27].

Pain may not be associated with this syndrome initially. It is only when postural imbalance continues for a long duration it can increase the load on neck and upper back muscles leading to discomfort and pain [3]. For patients who have been in pain for longer than three months, instrument-assisted soft tissue mobilization has been found to be more effective than traditional physical therapy methods. When moved over the skin it can effectively break down adhesions and cross-linkages and thus relieve the pain [23]. Short-term benefits of manual therapy were seen as there was no long-term follow-up post-intervention.

Myofascial release technique in which sustained pressure is applied over the restricted fascia helps to relieve pain and restoration of movement. This technique when combined with stretching exercises yielded better benefits than a myofascial release technique alone [17]. Compared to conventional stretching procedures, muscular energy approaches were more effective for both acute and chronic pain as a result of the increased flexibility of contractile and non-contractile tissue and the stimulation of proprioceptors and mechanoreceptors [20,24]. Regarding reducing pain and improving postural alignment, myofascial rollers outperformed post-isometric relaxation therapy [29].

The careful assessment of included articles using the Cochrane risk of bias tool is the review’s key strength. The results were interpreted despite discrepancies in study reporting. It was challenging to pool the data and evaluate the overall pooled estimate of effect due to the heterogeneity of included studies in terms of study design, type of intervention, duration, and outcome measure. Despite the variability, the meta-analysis was still considered appropriate because postural parameters were evaluated using the same photogrammetry technology in many of the studies, and a remedial exercise program was provided.

One of the limitations of the review was that the risk of bias was frequently high to moderate and unclear in many studies. Out of 18 studies included in the systematic review, six had a high risk of bias, four had a moderate risk, three had an unclear risk, and only five had a low risk of bias. This led to a reduced level of confidence and reliability of the findings.

The inclusion of research published in the English language increased the likelihood of language bias, which is one of the limitations of the study. A small sample size can affect the generalizability of the results. Additionally, none of the studies discussed the long-term effects of different interventions, which may also be a limitation. This hinders our ability to comprehend the sustainability and long-term implications of physiotherapy intervention.

## Conclusions

This comprehensive review and meta-analysis found that a multimodal approach combined with ergonomic intervention is useful in the treatment of upper cross syndrome. Exercise therapy was found to be beneficial in correcting postural alignment and movement patterns, while manual therapy was found to be similarly effective in pain reduction and functional impairment improvement. The findings were based on low to poor-quality evidence. Further good-quality research is warranted to explore the most effective physiotherapy intervention strategies for upper cross syndrome.

## Appendices

Appendix 1

**Table 2:** Search strategy.

Search strategy
Search Strategy for PubMed “upper crossed syndrome”[All Fields] OR “upper cross syndrome”[All Fields] # (“upper crossed syndrome”[All Fields] OR “upper cross syndrome”[All Fields]) AND “musculoskeletal*”[All Fields] # (“upper crossed syndrome”[All Fields] OR “upper cross syndrome”[All Fields]) AND “neck pain”[All Fields] #(“upper crossed syndrome”[All Fields] OR “upper cross syndrome”[All Fields]) AND (“manage”[All Fields] OR “managed”[All Fields] OR “managements”[All Fields] OR “managements”[All Fields] OR “manager”[All Fields] OR “managers”[All Fields] OR “managers”[All Fields] OR “manages”[All Fields] OR “managing”[All Fields] OR “management”[All Fields] OR “organization and administration”[MeSH Terms] OR (“organization”[All Fields] AND “administration”[All Fields]) OR “organization and administration”[All Fields] OR “management”[All Fields] OR “disease management”[MeSH Terms] OR (“disease”[All Fields] AND “management”[All Fields]) OR “disease management”[All Fields]) #“upper crossed syndrome”[All Fields] AND “upper cross syndrome”[All Fields] AND (“rehabilitant”[All Fields] OR “rehabilitants”[All Fields] OR “rehabilitate”[All Fields] OR “rehabilitated”[All Fields] OR “rehabilitates”[All Fields] OR “rehabilitating”[All Fields] OR “rehabilitation”[MeSH Terms] OR “rehabilitation”[All Fields] OR “rehabilitations”[All Fields] OR “rehabilitative”[All Fields] OR “rehabilitation”[MeSH Subheading] OR “rehabilitations”[All Fields] OR “rehabilitational”[All Fields] OR “rehabilitator”[All Fields] OR “rehabilitators”[All Fields]) # (“upper crossed syndrome”[All Fields] OR “upper cross syndrome”[All Fields]) AND (“interventions”[All Fields] OR “interventions”[All Fields] OR “interventive”[All Fields] OR “methods”[MeSH Terms] OR “methods”[All Fields] OR “intervention”[All Fields] OR “interventional”[All Fields] OR (“therapeutics”[MeSH Terms] OR “therapeutics”[All Fields] OR “treatments”[All Fields] OR “therapy”[MeSH Subheading] OR “therapy”[All Fields] OR “treatment”[All Fields] OR “treatments”[All Fields])) #(“upper crossed syndrome”[All Fields] OR “upper cross syndrome”[All Fields]) AND (“musculoskeletal manipulations”[MeSH Terms] OR (“musculoskeletal”[All Fields] AND “manipulations”[All Fields]) OR “musculoskeletal manipulations”[All Fields] OR (“manual”[All Fields] AND “therapy”[All Fields]) OR “manual therapy”[All Fields] OR “electrotherpy”[All Fields] OR (“exercise”[MeSH Terms] OR “exercise”[All Fields] OR “exercises”[All Fields] OR “exercise therapy”[MeSH Terms] OR (“exercise”[All Fields] AND “therapy”[All Fields]) OR “exercise therapy”[All Fields] OR “exercising”[All Fields] OR “exercises”[All Fields] OR “exercised”[All Fields] OR “exerciser”[All Fields] OR “exercisers”[All Fields])) #(“upper crossed syndrome”[All Fields] OR “upper cross syndrome”[All Fields]) AND (“physical therapy modalities”[MeSH Terms] OR (“physical”[All Fields] AND “therapy”[All Fields] AND “modalities”[All Fields]) OR “physical therapy modalities”[All Fields] OR “physiotherapies”[All Fields] OR “physiotherapy”[All Fields] OR (“physical therapy modalities”[MeSH Terms] OR (“physical”[All Fields] AND “therapy”[All Fields] AND “modalities”[All Fields]) OR “physical therapy modalities”[All Fields] OR (“physical”[All Fields] AND “therapy”[All Fields]) OR “physical therapy”[All Fields])) #(“upper crossed syndrome”[All Fields] OR “upper cross syndrome”[All Fields]) AND (“postural”[All Fields] OR “posturally”[All Fields] OR “posture”[MeSH Terms] OR “posture”[All Fields] OR “postures”[All Fields] OR “postured”[All Fields] OR “posturing”[All Fields]) #“upper crossed syndrome”[All Fields] OR “upper cross syndrome”[All Fields] AND “forward head posture”[All Fields] Search Strategy for Google scholar/Science direct #Abstract or title “upper crossed syndrome” OR “upper cross syndrome” #Abstract or title (“upper crossed syndrome” OR “upper cross syndrome”) and (“management”) #Abstract or title (“upper crossed syndrome” OR “upper cross syndrome”) and (“rehabilitation”) #Abstract or title (“upper crossed syndrome” OR “upper cross syndrome”) and (“Intervention”) #Abstract or title (“upper crossed syndrome” OR “upper cross syndrome”) AND (“treatment strategy”) #Abstract or title (“upper crossed syndrome” OR “upper cross syndrome”) AND (“manual therapy”) #Abstract or title (“upper crossed syndrome” OR “upper cross syndrome”) AND (“physiotherapy” OR “physical therapy”) #Abstract or title (“upper crossed syndrome” OR “upper cross syndrome”) AND (“musculoskeletal disorders”) #Abstract or title (“upper crossed syndrome” OR “upper cross syndrome”) AND (“corrections”) #Abstract or title Abstract or title (“upper crossed syndrome” OR “upper cross syndrome”) AND (“neck pain”) #Abstract or title (“upper crossed syndrome” OR “upper cross syndrome”) AND (“neck pain” OR “shoulder pain”) #Abstract or title (“upper crossed syndrome” OR “upper cross syndrome”) AND (“posture”) #Abstract or title (“upper crossed syndrome” OR “upper cross syndrome”) AND (“forward head posture”)

Appendix 2

**Table 3:** Summary of findings. CI = confidence interval; SMD = standardized mean difference. GRADE Working Group grades of evidence High certainty: We are very confident that the true effect lies close to that of the estimate of the effect. Moderate certainty: We are moderately confident in the effect estimate: the true effect is likely to be close to the estimate of the effect, but there is a possibility that it is substantially different. Low certainty: Our confidence in the effect estimate is limited: the true effect may be substantially different from the estimate of the effect. Very low certainty: We have very little confidence in the effect estimate: the true effect is likely to be substantially different from the estimate of effect.

Outcome	Number of participants	SMD (95% CI)	Risk of bias	Certainty of evidence
Craniovertebral angle	111	-2.24 (-4.83, 0.35)	High	⨁◯◯◯ Very low
Rounded shoulder	111	-1.42 (-2.94, 0.10)	High	⨁◯◯◯ Very low
Kyphotic angle	111	-1.69 (-2.98, -0.39)	High	⨁◯◯◯ Very low
Pain	160	-0.71 (-1.04, -0.39)	High	⨁⨁◯◯ Low
Functional improvement	220	-0.57 (-1.00, -0.14)	High	⨁⨁◯◯ Low
